# Understanding and preventing drug–drug and drug–gene interactions

**DOI:** 10.1586/17512433.2014.910111

**Published:** 2014-04-19

**Authors:** Cara Tannenbaum, Nancy L Sheehan

**Affiliations:** ^a^ Université de Montreal, Centre de Recherche de l’Institut universitaire de gériatrie de Montréal, 4565 Queen Mary Road #4824, Montreal, Québec H3W 1W5, Canada; ^b^ Université de Montréal, and Chronic Viral Illness Service, McGill University Health Centre, 3650 St. Urbain, D2.01, Montréal, Québec H2X 2P4, Canada

**Keywords:** cytochrome-mediated drug interactions, drug-drug-gene interactions, drug transporters, pharmacogenomics, polypharmacy

## Abstract

Concomitant administration of multiple drugs can lead to unanticipated drug interactions and resultant adverse drug events with their associated costs. A more thorough understanding of the different cytochrome P450 isoenzymes and drug transporters has led to new methods to try to predict and prevent clinically relevant drug interactions. There is also an increased recognition of the need to identify the impact of pharmacogenetic polymorphisms on drug interactions. More stringent regulatory requirements have evolved for industry to classify cytochrome inhibitors and inducers, test the effect of drug interactions in the presence of polymorphic enzymes, and evaluate multiple potentially interacting drugs simultaneously. In clinical practice, drug alert software programs have been developed. This review discusses drug interaction mechanisms and strategies for screening and minimizing exposure to drug interactions. We also provide future perspectives for reducing the risk of clinically significant drug interactions.

Research on receptor pharmacology, pharmacokinetics (PK) and pharmacodynamics (PD) historically involved single-drug approaches. Recognition that clinical adverse events could be caused by drug–drug interactions due to shared metabolic pathways arose in the late 1970s [1]. By 1990, reports of sudden death in patients taking terfenadine and ketoconazole contributed to the eventual withdrawal of terfenadine and other drugs from the US market [2]. Although some drug labels began to include the metabolic profile of drugs, systematic evaluation of drug interactions was not yet part of the formal drug approval process. The first guidance document to industry on the conduct of premarketing drug metabolism and drug interaction studies appeared in 1997 by the US FDA [3,4]. Since the publication of this document, understanding of the cytochromes and drug transporters has evolved and new methods have emerged to try to predict clinically relevant drug–drug interactions [1]. The use of freshly isolated or cryopreserved human hepatocytes or Caco-2 cells to evaluate isoenzyme and transporter expression is one such scientific advancement [1]. Another is the recognized need to identify the impact of pharmacogenetic polymorphisms on drug–drug interactions, and to assess interactions between more than two medications simultaneously [5].

Strategies to reduce drug interactions in clinical practice lag behind the initiatives taken during the drug preapproval process to predict and confirm drug interactions. Knowledge on potential drug interactions has primarily been translated to clinicians through the use of product monographs, health information technology and drug alert software programs. Three challenges exist for the efficient implementation of this knowledge. First, many drug interactions have been studied in healthy volunteers and the clinical outcomes have not been confirmed in clinical studies or practice. Predicted interactions do not always lead to discernible toxicity or therapeutic failure, thus confounding the need for intervention [6–8]. The fact that there is no consistent rating system to gauge the severity and likelihood of potential drug–drug interactions leads to a lack of consensus on decisions whether to change therapy [9,10]. Second, the pairwise interactions displayed by drug alert software programs are difficult to extrapolate to patients with complex drug regimens and polypharmacy. In practice, patients may receive multiple inhibitors of a given cytochrome or receive an inhibitor and an inducer of the same cytochrome, rendering the prediction of the clinical relevance of these interactions difficult. Further, because of the high frequency of two-drug alerts, physicians and pharmacists tend to override the majority of drug alert warnings [11,12]. Finally, any clinically significant drug interaction that is identified requires time and effort. If the pharmacist recognizes a potential drug–drug or drug–gene interaction, the physician must be notified and a management plan must be recommended, whether it be a modification in drug therapy or closer monitoring of efficacy and adverse drug reactions. In some countries, community pharmacists succeed in reaching physicians in only half the cases, and physicians do not always consent to changing prescriptions [13]. Even when physicians agree, additional steps must be taken to counsel the patient on the reasons why the prescription is being changed and to follow-up with the patient after the switch. Follow-up may consist of additional appointments, physical exams and repeat laboratory tests including in some cases therapeutic drug monitoring.

The purpose of this review is to examine drug interaction mechanisms and highlight the prevalence and importance of drug–drug and drug–gene interactions. Various strategies for identifying and preventing potential drug interactions will be discussed. During the coming era of cost containment in healthcare, it is likely that both policy and practice will increase emphasis on preventive approaches to curb adverse drug events and their associated costs.

## The CYP450 enzymes

The CYP450 enzymes are a superfamily of heme-containing microsomal enzymes whose main role during Phase I liver reactions is to oxidize, reduce or hydrolyze drug substrates to activate a prodrug or convert parent drugs to active or inactive metabolites to be eliminated. Food, environmental factors, other drugs and genetics influence cytochrome activity and subsequent drug metabolism [14]. Eight individual cytochromes with distinct but overlapping substrate specificities are widely recognized as being clinically relevant for drug metabolism: CYP1A2, CYP2B6, CYP2C8, CYP2C9, CYP2C19, CYP2D6, CYP3A4 and CYP3A5 [1]. Medications interacting with the CYP450 system can be classified as substrates, inhibitors or inducers. Inhibitors can be further characterized as being weak, moderate or potent [1].

## Transporters

Transporters of exogenous and endogenous substances across membranes may be divided into three large families: the solute carrier transporters that include eight protein subtypes [organic anion transporting polypeptides (OATPs), organic anion transporters, organic cation transporters, organic cation/carnitine transporters, peptide transporters, concentrative nucleoside transporters, equilabrative nucleoside transporters and multidrug and toxin extrusion transporters]; the ATP-binding cassette transporters comprising multidrug resistance protein (p-glycoprotein or MDR1), multidrug resistance associated proteins and breast cancer resistance protein; the bile acid, cholesterol, aminophospholipid and copper transporters [15–17]. Tissue distribution of these transporters is widespread, including enterocytes, hepatocytes, renal tubule epithelial cells, the blood–brain barrier and the placenta [15]. For each solute carrier transporter and ATP-binding cassette transporter, numerous medications have been identified as substrates and may be vulnerable to inhibition or induction [17]. Though the substrates of the third family of transporters are mainly endogenous products, certain medications may also be substrates or inhibitors of these transporters. These drug–transporter interactions may be clinically relevant; in particular, the recent demonstration that medications that are potent bile salt export pump inhibitors are more likely to cause drug-induced liver injury [18].

## Mechanisms of drug–drug interactions

Drug–drug interactions may be divided into PD and PK interactions. PD interactions occur when medications cause additive or antagonistic pharmacological effects influencing efficacy or adverse effects. The administration of warfarin and nonsteroidal anti-inflammatory drugs is an example of a PD interaction as their concomitant use can increase the risk of bleeding [19]. PK interactions can be due to changes in absorption, distribution, metabolism and elimination. The following section focuses on the mechanisms of PK drug interactions. Selected examples of clinically significant drug–drug interactions are provided.

### Absorption

Absorption-related drug interactions are commonly associated with three distinct mechanisms. First, for certain medications, decreased absorption may be secondary to chelation with a cation such as calcium or iron. For example, the simultaneous administration of ferrous sulfate with ciprofloxacin decreases ciprofloxacin area under the concentration-time curve (AUC) and maximum concentration (C_max_) by 57 and 54%, respectively [20]. This interaction may potentially lead to therapeutic failure and the development of resistance. Second, absorption may be decreased when dissolution of the medication is highly dependent on gastric pH. Atazanavir, an HIV protease inhibitor, requires low gastric pH to be absorbed and will be influenced by gastric acid-modifying agents. For instance, administration of omeprazole with ritonavir boosted atazanavir decreases atazanavir exposure by 42% [21]. This decrease in exposure may be clinically significant for patients with partially resistant HIV, increasing their risk of virological failure. Third, intestinal absorption may be influenced by inhibition or induction of CYP450 enzymes (overwhelmingly CYP3A4) or the p-glycoprotein efflux transporter in the intestinal epithelium [22]. Metabolism of cyclosporine has been demonstrated to take place both in the gut and in the liver. Potent inducers or inhibitors of CYP3A such as rifampin or erythromycin increase or decrease gut extraction of cyclosporine, respectively [23]. This may potentially increase the risk of graft rejection in the former case or of nephrotoxicity and other adverse drug reactions in the latter case.

### Distribution

Distribution of medications into tissues is mediated by drug influx and efflux transporters and influenced by protein binding as only the free fraction will be able to penetrate across tissue membranes. In addition to p-glycoprotein-associated interactions in the gut, some clinically significant drug interactions are associated with other transporters such as rosuvastatin and cyclosporine via OATP1B1 in hepatocytes. In patients receiving cyclosporine post heart transplant, rosuvastatin AUC and maximum concentration was increased 7.1- and 10.6-fold, respectively, compared with historical controls on rosuvastatin without cyclosporine [24]. The mechanism was confirmed by an *in vitro* study showing that rosuvastatin uptake in the hepatocyte by OATP1B1 is inhibited by cyclosporine [24]. Though cases of myopathy were not seen in this study, a potential increased risk of rhabdomyolysis remains. A lingering concern is also the risk of decreased efficacy of rosuvastatin if it cannot enter the hepatocyte where it is active.

Drug–drug interactions mediated by protein binding displacement are probably not clinically significant. With protein binding displacement, the total concentration is often lower, but the concentration of the free medication remains relatively unchanged. This is explained by an increased clearance of the unbound fraction. As the concentration of the unbound (active) medication is similar, no decrease in efficacy or important toxicity is expected. This explains why despite a 29 and a 40% decrease in R-methadone AUC and minimum concentration with the coadministration of methadone and telaprevir, a hepatitis C protease inhibitor, patients did not present any opiate withdrawal symptoms [25].

### Metabolism

Metabolic interactions are mostly due to CYP450 isoenzymes [26]. Table 1 illustrates potentially harmful two-drug cytochrome-mediated interactions that are well recognized and easy to predict [27]. The examples represent a sample of drug interactions that are associated with increased morbidity, hospitalization or mortality. Phase II metabolic reactions, or conjugation, may also be implicated in drug–drug interactions, in particular glucuronidation. The transfer of glucuronide acid moieties to molecules by uridine diphosphate glucuronosyltransferases (UGTs) can be inhibited or induced. UGT1A1, 1A3, 1A4, 1A6, 1A7, 1A8, 1A9, 1A10 and 2B4, 2B7, 2B15, 2B17 and 2B28 have been associated with glucuronidation of medications [28]. Multiple clinically significant drug–drug interactions are due to inhibition or induction of glucuronidation. For example, valproic acid, by inhibiting UGT2B7, increases zidovudine AUC twofold [29]. This may cause an increased risk of anemia [30]. Valproic acid, by the same mechanism, also increases lamotrigine AUC by 309%, potentially explaining the increased risk of rash when these two medications are coadministered [31–33]. In opposition, rifampin, a potent inducer of glucuronidation through pregnane X receptor and constitutive androstane receptor induction, decreases lamotrigine and zidovudine AUC by 44 and 47%, respectively, potentially leading to therapeutic failure [28,34,35].

**Table 1.** **Some examples of cytochrome-mediated drug interactions that increase morbidity, hospitalization and mortality.**

**Cytochrome**	**Interactions**	**Type and magnitude of effect**	**Ref.**
1A2	Theophylline + ciprofloxacin (1A2 inhibitor)	The coadministration of theophylline with ciprofloxacin, compared with other antibiotics, increases the risk of hospitalization due to theophylline toxicity by almost twofold (adjusted OR: 1.86, 95% CI: 1.18–2.93)	[98]
2C8	Repaglinide + gemfibrozil (2C8 inhibitor)	Gemfibrozil increases repaglinide AUC and C_max_ by 812 and 240%, respectively. Patients have a greater risk of hypoglycemia when receiving gemfibrozil	[99]
2C9	Warfarin + Trimethoprim/ sulfamethoxazole (TMP/SMX) (SMX 2C9 inhibitor)	The use of TMP/SMX increases the risk of hospitalization due to upper gastrointestinal bleeding fourfold in patients on warfarin (OR: 3.84, 95% CI: 2.33–6.33). An increased risk of hospitalization for gastrointestinal bleeding in warfarin users occurs within 6-10 days of using TMP/SMX compared with other agents (OR: 1.68, 95% CI: 1.21–2.33)	[57,58]

2C9	Phenytoin + TMP/SMX (SMX 2C9 inhibitor)	Twofold higher risk of hospitalization for phenytoin toxicity with use of TMP/SMX in prior 30 days compared with amoxicillin (OR: 2.11, 95% CI: 1.24–3.60)	[59]
2C9	Glyburide + TMP/SMX (SMX 2C9 inhibitor)	Elderly patients receiving glyburide admitted with hypoglycemia were 6-times more likely to have been treated with TMP/SMX in the previous week (OR: 6.6, 95% CI: 4.5–9.7). The use of TMP/SMX increases the risk of severe hypoglycemia in glyburide users compared with cephalosporins (OR: 2.68, 95% CI: 1.59–4.52)	[60,61]
2D6	Tamoxifen + paroxetine (2D6 inhibitor)	An increased risk of death is associated with concomitant use of tamoxifen and paroxetine in women older than 65 years being treated for breast cancer	[47]
3A4	Nifedipine + erythromycin or clarithromycin (3A4 inhibitors)	In elderly patients taking calcium channel blockers, erythromycin coadministration within the previous 7 days is most strongly associated with hospitalization due to hypotension (OR: 5.8; 95% CI: 2.3–15.0), followed by clarithromycin (OR: 3.7, 95% CI: 2.3–6.1)	[62]
3A4	Oral contraceptives (ethinyl estradiol and norethindrone) + rifampin (3A4 inducer)	Rifampin decreases ethinyl estradiol AUC by 64%. Unplanned pregnancies have been reported when rifampin is given with oral contraceptives	[100,101]
3A4	HMG-CoA reductase inhibitors (statins) (atorvastatin, simvastatin and lovastatin) + erythromycin or clarithromycin (3A4 inhibitors)	In a geriatric population, coadministration of a statin with erythromycin or clarithromycin, compared to azithromycin, was associated with an increased risk of hospitalization due to rhabdomyolysis (RR: 2.17, 95% CI: 1.04–4.53) or acute kidney injury (RR: 1.78, 95% CI: 1.49–2.14). Patients on erythromycin or clarithromycin also had an increased risk of mortality (RR: 1.56, 95% CI: 1.36–1.80)	[102]
3A4	Intra-articular triamcinolone + ritonavir (3A4 inhibitor)	Fifteen case reports of iatrogenic Cushing syndrome with suppression of the hypothalamic–pituitary–adrenal axis associated with intra-articular injections of corticosteroids (primarily triamcinolone) have been reported in patients receiving ritonavir	[103]

AUC: Area under the concentration–time curve; C_max_: Maximum concentration; OR: Odds ratio; RR: Relative risk.

### Elimination

The inhibition of tubular secretion of a medication by a perpetrator drug has long been recognized as an important drug interaction mechanism. A better description of the role of influx and efflux transporters in renal cells has further enhanced our understanding of specific mechanisms influencing elimination. For example, clarithromycin decreases digoxin renal secretion through inhibition of p-glycoprotein in the kidney cells [36].

In another example, gemfibrozil inhibits OAT3 transporter-mediated renal clearance of pravastatin, increasing pravastatin exposure twofold as well as the risk of creatine kinase elevations [37–39].

## Drug–gene interactions

Important genetic polymorphisms exist for CYP2C9, CYP2C19 and CYP2D6, accounting for a substantial portion of person-to-person variability in drug metabolism [40]. The distribution of each polymorphism differs according to ethnicity. For example, approximately 10% of the white population and 1% of the Asian and black populations are poor metabolizers of CYP2D6 drugs, whereas 5–10% of the white population are ultra-metabolizers [41].

A better understanding of the impact of interindividual differences in the metabolic capacity of polymorphic cytochrome isoenzymes is rapidly evolving [42]. Zangar *et al*. investigated the top 200 drugs most often prescribed in the USA in 2008 and found that members of the CYP3A family contributed to the metabolism of 37% of the drugs, followed by CYP2C9 (17%), CYP2D6 (15%), CYP2C19 (10%) CYP1A2 (9%) and CYP2C8 (6%) [43]. The results suggest that the clinically well-established polymorphisms of CYP2C9, CYP2C19 and CYP2D6 may be relevant in almost half of the top 200 drugs prescribed. Commonly implicated drugs include NSAIDs metabolized by CYP2C9, proton-pump inhibitors metabolized by CYP2C19, and β-blockers and several antipsychotics and antidepressants metabolized by CYP2D6. To date, the literature supporting the impact of certain polymorphisms on clinical outcomes ranges from extensive to insufficient for different drugs [42]. For instance, the risk of bleeding with clopidogrel is reduced in carriers of CYP2C19-deficient alleles, with the risk of cardiovascular events moderately increased [44]. Women with CYP2D6-deficient polymorphisms have increased breast cancer recurrence rates under tamoxifen therapy [45]. Pain relief is compromised with the use of codeine in slow metabolizers of CYP2D6, while the risk of CNS toxicity and respiratory depression is higher in ultrarapid metabolizers due to excessive conversion to morphine [46]. These effects may be amplified in the presence of drug–drug interactions [42,47].

UGTs are also subject to polymorphisms, UGT1A1*28 being perhaps the most well-known polymorphic allele and associated with unconjugated hyperbilirubinemia (Gilbert’s syndrome) [28,48]. Drug–UGT gene interactions can increase the risk of adverse drug reactions, such as increased atazanavir-associated hyperbilirubinemia or irinotecan-related toxicity in patients with Gilbert’s syndrome [49–51]. Further, a multitude of drug transporter polymorphisms have been identified, certain leading to an increased risk of adverse effects such as renal proximal tubulopathy in patients with specific multidrug resistance associated proteins 2 haplotypes receiving tenofovir [17,52].

## Influence of genetic polymorphisms on drug–drug interactions

Beyond simply considering drug–drug interactions and drug–gene interactions, we now know that many genetic polymorphisms can influence the expression of drug–drug interactions. This is particularly the case when multiple metabolic pathways are involved, as is the case with the voriconazole–atazanavir/ritonavir interaction. Voriconazole is primarily metabolized by CYP2C19 and less so by CYP3A4 and CYP2C9 [53,54]. When atazanavir/ritonavir is coadministered, voriconazole AUC decreases by 33% in patients who are extensive metabolizers for CYP2C19 (as ritonavir induces CYP2C19) but increases voriconazole AUC by 461% in patients who are CYP2C19 poor metabolizers (as atazanavir/ritonavir are potent CYP3A4 inhibitors) [55]. These two populations need very different doses of voriconazole, 200–300 mg BID for extensive metabolizers and 50 mg BID for poor metabolizers. Hence, without pharmacogenetic testing, clinicians cannot predict the extent of the interaction nor how to dose voriconazole, leading to potential toxicity or inactivity.

Similarly, drug interactions can influence phenotypic expression, a process called phenoconversion. For example, the presence of a CYP450 inhibitor or inducer can change a person’s phenotype from a nonpoor metabolizer to a poor metabolizer or vice versa, as occurs in patients with depression receiving venlafaxine and CYP2D6 inhibitors [56].

## Clinical significance of drug–drug interactions

The clinical significance of an interaction will depend on several factors, including the PK/PD relationship and the therapeutic index of the victim drug, the potency and concentration of the inhibitor or inducer, the proportion of the victim drug affected by the specific metabolic, elimination or transport pathway that is inhibited or induced, the baseline bioavailability of the victim drug, whether the victim drug is a prodrug or an active drug, pharmacogenomics and the effects of disease on other PK and PD parameters.

Ultimately, a drug interaction should be considered clinically significant if patients have modified efficacy or increased adverse effects. Few drug–drug interaction studies, however, are conducted in patient populations to evaluate therapeutic outcomes or are long enough to completely assess the development of adverse effects. Population cohort studies are perhaps the best design to evaluate outcomes such as prevalence of adverse effects, treatment discontinuations, hospitalizations and mortality as demonstrated in Table 1 [27,47,57–63].

In PK drug–drug interaction studies, the common method used for determining whether a drug–drug interaction is clinically significant is the use of a no effect boundary of 80–125%. With this approach, if the 90% confidence intervals of the geometric mean ratio of the AUC (test vs reference) are contained completely between 80 and 125%, the interaction is considered not clinically significant. This default no effect boundary, however, may sometimes be inappropriate. The no effect boundary for a given drug should be individualized, whenever possible, with the exposure–response data (or PK/PD relationship) [64]. For example, for certain medications a 30% drop in concentration is not clinically significant, whereas it may lead to therapeutic failure with other medications with a narrower therapeutic index. The clinical significance of an increase or decrease in plasma concentration of a medication will be greatest for drugs with a narrow therapeutic index. Some examples of narrow therapeutic index drugs include theophylline (CYP1A2), paclitaxel (CYP2C8), warfarin (CYP2C9), phenytoin (CYP2C19) and the CYP3A4 substrates cyclosporine, dihydroergotamine, fentanyl, quinidine, pimozide, sirolimus and tacrolimus [64]. These medications are associated with serious toxicity if the exposure is increased, such as major bleeding with warfarin and respiratory depression with fentanyl, and require careful dose titration and close monitoring.

## Prevalence & risk of drug–drug interactions

Consensus panels differentiate between potential and actual drug–drug interactions [9]. A potential drug interaction is an occurrence in which two drugs known to interact are concurrently prescribed, regardless of whether adverse events occur. An actual drug interaction is an alteration in a clinically meaningful way of the effect of an object drug as a result of coadministration of another drug (precipitant drug). Potential drug interactions necessarily antecede actual drug interactions. One strategy to minimize the risks associated with potentially harmful drug combinations is to reduce exposure to concurrent administration. However, this is not always feasible when the benefits for a given patient outweigh the risks or if substitutions are unavailable.

The probability of any drug interaction logically increases as a function of the number of drugs consumed [65]. Drug interactions will therefore occur with greatest frequency in the presence of polypharmacy and will be more likely when specific medications depend on CYP450 metabolism for their activation or elimination. These two risk factors are most common in the elderly, in patients with multimorbidity, and in specific subgroups of individuals who are more likely to take certain clusters of medications, such as patients with psychiatric conditions or those requiring antimicrobial agents [10,27,62,66–69]. In Canada, among patients aged 65 years and older with polypharmacy (>5 drugs) admitted to hospital, the prevalence of potential cytochrome-mediated drug interactions is reported to be 80% [10,66]. This estimate exceeds the 73 and 68% prevalence of interactions reported on general adult and geriatric psychiatry units, respectively, in the UK [67]. The probability of detecting at least one interaction varies with the number of drugs consumed and is expected to be 50% for persons taking 5–9 drugs, 81% with 10–14 drugs, 92% with 15–19 drugs and 100% with 20 drugs or more [66]. The implication of detecting a large number of drug interactions requires further study.

Ninety-three percent of potential cytochrome-mediated drug interactions in the elderly can be attributed to concomitant administration of drugs metabolized by CYP3A4 or CYP2D6, with 70% attributable to CYP3A4 alone [10]. A cross-sectional study of 900 patients from six different populations in the Netherlands demonstrated that geriatric, psychogeriatric and psychiatric patients present a twofold higher risk of being treated with at least one drug metabolized by CYP2D6 compared with the general population [68]. Various authors have highlighted the common occurrence of potential CYP3A4 and CYP2D6 interactions in other populations ranging from the critically ill patient to the outpatient dermatology patient [14,69]. However, the most prevalent drug interactions may not necessarily be the most severe [70]. Malone *et al*. published consensus ratings from a US expert panel on the clinical importance of 56 different drug–drug interactions seen in community and ambulatory pharmacy settings [70]. Of these, only half were deemed clinically important, involving drugs such as monoamine oxidase inhibitors, amiodarone and azole antifungal agents. The panel noted substantial gaps in the literature on the quality of the evidence for substantiating the severity of many *in vitro* drug interactions in practice, with only moderate consensus achieved on the final list of ratings. In 2013, Andersson *et al.* looked at the frequency of potentially severe PK drug interactions with warfarin in the adult Swedish population using a national registry and found the prevalence to be quite low; per 1000 warfarin users only 7.8 took carbamazepine, 4.0 were using sulfamethoxazole and 3.7 filled a prescription for fluconazole [71].

## Strategies to reduce the risk of drug interactions

In order to detect patients at risk for harmful drug interactions, potential drug interactions must first be identified. Strategies to reduce the risk of interactions encompass regulatory endeavors to improve labeling on the metabolic profile of new drugs as well as potentially hazardous drug–drug and drug–gene combinations. A number of software programs for identifying and managing potential drug interactions are also available. Pharmacogenetics is becoming increasingly available in many countries. Ultimately, however, decision aids for prescribing are only as good as the individuals who make judicious use of them. Implementation of quality indicators such as population surveillance for clinically significant drug interactions [72], with audit and feedback to the dispenser and prescriber, may be the leap that is required to propel drug interaction management into mainstream medicine.

### Regulatory requirements & drug labeling

The past 5 years have seen new documents released by the US FDA and the International Conference on Harmonization to improve the conduct of PK studies in older adults and in patients with polypharmacy [5,73]. These guidelines provide detailed recommendations for industry regarding the *in vitro*, *in vivo* and clinical trial evaluation of drug metabolism and drug transporter interactions. The goal of the new guidelines is to provide useful information in the product label to narrow the gap between what is known at the time of approval of specific drugs and the risk of serious effects in the longer term, particularly in high-risk complex populations such as those with multimorbidity and polypharmacy.

To be clinically helpful, PK drug–drug interaction data in product labels should be accompanied by clear recommendations for clinicians for managing and monitoring the interactions. The data to support recommendations about drug combinations listed as boxed warnings or contraindications should also be provided and should be consistent with other data sources [74–76]. In the USA, there is some concern that clinically irrelevant and/or unsubstantiated warnings in product labeling [77] contribute to alert fatigue or serve merely to protect against liability, an approach that may undermine identification of truly clinically relevant interactions in clinical practice [78].

Few studies have described the effects of previous regulatory guidance on drug labeling [79,80]. Marroum *et al*. report that of the 540 drug–drug interaction studies conducted during the mid-1990s, only 15% resulted in clinically significant labeling statements. One percent of these statements included recommendations for monitoring and 4% for a labeled contraindication [79]. Drug labeling is a complex issue that requires balance between clinical relevance, consistency of information and substantive data quality. The effect of newer guidelines on product labeling changes for older patients and patients with polypharmacy has yet to be determined.

### Pharmacogenotyping

There is an emerging interest on the part of clinical pharmacologists, clinicians and patients to be able to predict a patient’s metabolizer phenotype to help direct the choice of therapy. As an example, in 2012, the Clinical Pharmacogenetics Implementation Consortium issued recommendations for CYP2D6 genotyping for patients requiring pain control; these guidelines were updated in 2014 [46]. Codeine is metabolized by CYP2D6 to its active metabolite and a growing body of evidence links CYP2D6 genotype to variability in codeine efficacy and toxicity. As the incidence of poor and ultrarapid CYP2D6 metabolizers varies between 0–10% and 0–29%, respectively, across various populations, guidelines were developed for codeine administration in the context of a patient’s CYP2D6 genotype [46]. As yet there have been no randomized trials involving pharmacogenetic testing to test the efficacy of the codeine administration guidelines. Another caveat is that CYP2D6 genotyping is reliable when performed in qualified laboratories. However, as with any laboratory test, a possible area of risk is an error in genotyping that could have adverse health implications for the patient.

Another area where genotyping has evolved is in patients requiring warfarin dosing [42]. Genotyping of *CYP2C9* in combination with the vitamin K epoxide reductase complex subunit 1 (*VKORC1*) gene, however, explains only 35% of the variability in the therapeutic warfarin dose, with other factors such as age, race, drug interactions and smoker status also influencing interindividual variability [81,82]. One of the obstacles with using clinical pharmacogenetic testing for warfarin dosing is the conflicting data as to whether genotyping significantly influences the time to achievement of the first therapeutic response and the risk of over-anticoagulation [42,83]. In a recent trial, an approach including pharmacogenotyping of *CYP2C9* and *VKORC1* to tailor warfarin dosing was superior to standard of care. Subjects with pharmacogenotyping were more likely to have an international normalized ratio in the therapeutic range, for a greater proportion of time, and have less serious adverse effects [84]. In 2011, the Clinical Pharmacogenetics Implementation Consortium published their guidelines for the interpretation of *CYP2C9* and *VKORC1* genotypes and the use of this tool in clinical practice to improve patient outcomes [85]. Currently, drug regulatory agencies do not require genotyping before warfarin initiation.

There are a growing number of publications on recommendations for the management of drug–gene interactions, but very little guidance as to how to interpret and manage drug–drug–gene interactions in the clinic. The recent understanding that genetic polymorphisms can influence the clinical significance of drug–drug interactions increases the complexity of managing these interactions. For example, how to manage a person who is a CYP2D6 ultrarapid metabolizer and who is receiving codeine and paroxetine, a potent CYP2D6 inhibitor? Other barriers include limited access to pharmacogenetic tests in hospitals and outpatient settings and insufficient knowledge of healthcare professionals related to pharmacogenomics [86,87]. This underlines the importance for industry and clinical pharmacologists to conduct drug–drug–gene interaction studies, for universities to include pharmacogenomics in their curriculum and for healthcare services to upscale access to pharmacogenetic testing.

### Drug interaction alert software & other resources

Drug interactions are listed in product monographs, compiled in pharmaceutical compendia, and are available in a number of books, websites and other resources; however, this information is not always easily accessible at the time of prescribing. To make drug interaction information more usable, computerized adverse drug event surveillance systems have been developed in the form of clinical decision-support software and are available in most hospitals and community practices, and downloadable as applications on handheld devices. Although these systems may augment clinicians’ ability to detect clinically significant interactions, these systems are far from fail-safe, often missing important interactions, eliciting alert fatigue and dismissal, and are prone to database inconsistencies [88–91]. Software programs that only evaluate two drug profiles at a time are unable to assess multidrug combinations simultaneously, leaving clinicians to rely on incomplete information to minimize multidrug interactions in patients with polypharmacy. Few software integrate information on drug–gene and drug–drug–gene interactions. The clinical context in which potential interactions occur is another factor that is being considered in the development of computerized physician order entry systems [92]. The clinical status and comorbidities of the patient, the professional experience of the user and the severity of the drug interaction effect are all being examined as critical elements for efficiently tailoring transmission of drug interaction information [92,93].

In the authors’ opinion, an ideal drug interaction alert software should interface with the patients’ electronic medical or pharmacy chart to rapidly and efficiently link drug interaction data with the patients’ full medication and pharmacogenotypic profiles and alert the clinician when interactions are detected; be able to assess multidrug interactions, drug–gene and drug–drug–gene interactions; focus not just on cytochrome-mediated interactions but also on drug–drug interactions that are secondary to other metabolic processes, transporters or that are of a PD nature; provide the clinician with an up-to-date summary of the quality of the evidence supporting the mechanism and clinical significance of the drug interaction, including prospective cohort data, case reports, PK drug interaction studies, PK/PD relationships, detailed PK characteristics of the medications including *in vitro* data on substrates, inhibitors and inducers; specify the severity and onset of the drug interaction and include clear management recommendations.

At the present, few drug interaction alert software have all these capacities. One example is the Intermed-Rx application [94]. This application presents in a one-page integrative color-coded matrix all potential multidrug interactions. The matrix lists all object and precipitant drugs metabolized by the same cytochrome and also reports noncytochrome-mediated drug interactions. Management recommendations are suggested based on clinical and PK drug interaction data and references. Sound clinical judgment is still required, but performing a single multidrug assessment rather than multiple, sequential two-drug assessments for patients with polypharmacy may reduce drug alert dismissal and increase appropriate risk reduction interventions [10,66]. Comparative performance evaluation of the multidrug software against a typical two-drug alert software program revealed detection of an average of three additional cytochrome-mediated drug interactions per patient with polypharmacy [66].

Drug interaction alert software, however, are not always adapted to specialized fields where drug interaction data are often presented at expert meetings. In this case, clinicians should review the available software and resources and choose 2–3 high-quality resources that are best adapted to their needs. For example, in the field of HIV where managing clinically significant drug–drug interactions is frequent and complex and where practice evolves rapidly, numerous specialized drug–drug interaction websites have been created and have been evaluated using criteria such as content, reliability, access restrictions and ease of navigation [95]. Finally, although drug decision support is believed to offer a solution for the prevention of drug–drug interactions, data from randomized trials have not yet conclusively showed that provision of this information to prescribers effectively reduces prescribing problems [96].

### Guidelines for prevention of drug interactions

To date there exists no computerized clinical decision support system that possesses the capacity to gauge whether potential drug interactions will yield severe, moderate or minimal clinical effects in any given patient. Users of clinical decision support systems are not only stymied by this lack of patient-specific information, but may be frustrated by their inability to differentiate clinically significant versus nonclinically significant interactions and to know when to intervene [91]. A major barrier to integration of this information in drug alert software is the quality, grading and synthesis of drug–drug interaction evidence. Recent recommendations have been published to improve the drug–drug interaction evidence base, to develop and promote a systematic approach for evaluating the evidence, and to integrate this evidence into meaningful clinical decision support systems to help clinicians judge when interventions are required [9]. Checklists for standardized reporting of drug–drug interaction management guidelines have also been developed [97]. In the meantime, clinicians can refer to prior classification systems that are sometimes included in drug alert software programs, but that are far from perfect. For instance, a drug interaction may have a clinical relevance of A (minor interaction, not significant), B (the outcome is uncertain or may vary), C (the interaction can be handled by dose adjustment) or D (the combination is best avoided) [9]. In Sweden, population surveillance of drug interactions is based on D classification only [72].

Until such time as the evidence base improves, the cautious clinician would do well to substitute safer medication alternatives whenever possible to prevent potential drug–drug interactions. Suggestions for managing cytochrome-mediated drug–drug interactions in the elderly have been published [10]. Many opportunities exist for substitution of drugs with the same therapeutic indication or within the same drug class that are metabolized by different isozymes or via separate metabolic pathways. However, in other instances, substitutions may not be possible, and a dosing or schedule adjustment may minimize potential interactions. In certain cases, the benefit of continuing concomitant administration of two interacting drugs may outweigh the risks. Risks may include destabilizing disease control, introducing new adverse drug reactions or increasing the risk of medication errors. Ideally the potential for drug–drug interactions should be discussed with each patient to enable monitoring of early clinical consequences. For patients whose genotype is known, guidelines can be followed for certain medications, such as warfarin and codeine administration [46,85]. In the cases of polypharmacy and multimorbidity, whenever the resources are available, pharmacists or clinical pharmacologists should be consulted to do a complete assessment of the drug interaction risk for a given patient, to offer recommendations to limit these risks and to arrange subsequent patient monitoring.

Ultimately increased awareness of drug–drug, drug–gene and drug–drug–gene interactions are the first step towards reducing exposure and minimizing the risks associated with potentially harmful drug combinations. Use of clinically useful, interactive, multidrug software and advances in pharmacogenotyping are important tools to help facilitate this process.

## Expert commentary

As rates of polypharmacy rise in concert with increasing life expectancy and multiple morbidities in the same individual, the authors expect that unnecessary costs associated with drug–drug interactions will lead to more pronounced efforts to minimize risk. De-prescribing in the context of polypharmacy is a relatively new concept propagated by geriatric pharmacists that is expected to gain popularity in coming years. More research in this area, such as how to decrease the variability of database rating systems, tiering of alerts, improving the identification of clinically significant alerts and increasing the patient specificity of the generated drug interaction alerts, should be conducted. Strategies to avoid alert fatigue are other areas for future study.

## Five-year view

As new evidence reveals the unrecognized financial burden of drug interactions on health system utilization, regulatory bodies may decide to encourage or even enforce preventative prescribing practices among primary care practitioners and pharmacists. The latter will require reinvestment in the time healthcare providers are allowed to spend on individual-based health assessments, as opposed to the factory-line efficiency currently demanded by cost-conscious managers. Furthermore, as genomic profiling becomes more accessible to the patient, patients may start to demand greater transparency and more safety reassurance from their prescribers. Therapeutic drug monitoring may increase. With changes in product labels and black-box warnings issued more and more frequently, the context of prescribing is bound to change.

Increasing awareness of the prevalence of potential drug–drug and drug–gene interactions, combined with judicious implementation of new regulatory requirements for industry to test for, classify and report drug interactions, will hopefully lead to an upstream shift in priorities for healthcare professionals to identify and prevent drug–drug interactions. In 5 years’ time, healthcare systems in developed countries may want to monitor and track polypharmacy indicators that include the prevalence of drug–drug interactions, both for patient safety and healthcare spending purposes. We hope that the combined efforts of clinical pharmacologists, clinicians and government to build momentum around the prevention of drug interactions will ultimately lead to better health outcomes for patients.

## Notes

Key issues

- The prevalence of potential drug–drug interactions is expected to rise in parallel with increasing rates of multimorbidity and polypharmacy in the general population.
- A growing understanding of the importance of preventing potential drug–drug and drug–gene interactions has led to changes in the regulatory requirements for industry to test for, classify and report interactions.
- Postmarketing studies and pharmacoepidemiological research have uncovered common and potentially harmful drug interactions that have important financial and health implications for the individual and society.
- Advances in clinical decision support systems may allow the evaluation of potential multidrug interactions while circumventing the alert fatigue elicited by repetitive two-drug alert pop-ups in traditional drug alert software.
- As pharmacogenotyping becomes more mainstream, both patients and their healthcare providers will increasingly come to rely on evidence-based published guidelines on how to avoid potential drug–gene and drug–drug–gene interactions.
